# Policy text analysis on inheritance and innovation of Mongolian medicine culture in China

**DOI:** 10.3389/fpubh.2026.1814485

**Published:** 2026-04-21

**Authors:** Shenzhen Liu, Zili Yang, Alun, Min Li

**Affiliations:** 1 School of Health Management, Inner Mongolia Medical University, Hohhot, China; 2 Innovation Center of Ethnic Medicine, Inner Mongolia Medical University, Hohhot, China

**Keywords:** cultural heritage sustainability, health industry ecosystem, policy text analysis, public health policy, traditional Chinese medicine (Mongolian medicine)

## Abstract

**Objective:**

To analyze the focuses and shortcomings of policies for the inheritance of and innovations in traditional Chinese medicine (Mongolian medicine) culture in China and to provide a reference for the subsequent optimization of relevant policies.

**Methods:**

The classification method of policy tools proposed by Rothwell and Zegveld was adopted. The policy objective dimensions were integrated, Nvivo15 software was used to conduct a coding analysis of 30 policy texts in the field of the inheritance of and innovations in traditional Chinese medicinal (Mongolian medicinal) culture from 2016–2024, and a two-dimensional analysis framework was constructed on the basis of policy tools and objectives.

**Results:**

In the X dimension (policy tools), supply-type policy tools accounted for the greatest proportion (40.92%), followed by the environment type (39.28%), and the demand type accounted for the least (19.80%). In the Y dimension (policy objectives), the construction of talent teams accounted for the greatest proportion (25.63%). Relatively fewer policy tools were available regarding the collation of ancient medical books and theoretical innovation research (6.88%) and standardization projects (6.25%).

**Conclusion:**

The configuration of policy instruments is structurally imbalanced, showing a “supply-prioritized and demand-neglected” pattern, with insufficient support for basic policy objectives (e.g., financial support, standardization projects). Implementation Suggestions A phased framework with explicit time nodes and prioritized steps is proposed for policymakers: [1] Short-term horizon (1–2 years): Filling supply-side gaps by increasing financial support to 8–10% (focusing on Mongolian medicine ancient document collation and digital platforms) and launch demand-side pilots (e.g., integrating into regional medical insurance, establishing 2–3 inheritance demonstration bases). [2] Medium-term (3–5 years): Optimize supply-side structure, raise demand-side instrument proportion to 25–30%, and promote the standardization of Mongolian medicine hospital preparations. [3] Long-term (more than 5 years): Establish a dynamic matching matrix between policy instruments and objectives, conduct annual evaluations, and improve multi-departmental collaboration. Prioritized steps: supplement financial gaps → launch demand-side pilots → optimize supply structure and promote standardization → establish long-term adjustment mechanisms. These measures aim to maximize policy effectiveness and promote the sustainable inheritance and innovation of Mongolian medicine culture.

## 1 Introduction

The global evolution of policies for traditional medicines (TMs) and integrated health systems reflects a profound shift from historical marginalization to formal recognition, regulation, and integration within national healthcare frameworks (1–5). This transformation has been largely driven by the World Health Organization’s (WHO) Traditional Medicine Strategies, associated World Health Assembly (WHA) resolutions, national policy reforms, and a growing body of evidence supporting the safety, efficacy, and cultural relevance of TMs (1–3). The WHO has played a pivotal role in this evolution, launching its first global strategy on traditional and alternative medicine in 2002 (6, 7). Subsequent strategies, including the WHO Traditional Medicine Strategy 2002–2005 and the 2014–2023 strategy, have aimed to enhance the contribution of traditional, complementary, and integrative medicine (TCIM) to the achievement of universal health coverage (UHC) (2–4, 8, 9). The current strategy (2014–2023) focuses on several key priorities: building a robust evidence base through research, establishing regulatory mechanisms to ensure quality and safety, and strengthening the integration of evidence-based TCIM into national health systems (3). The WHO Global Traditional Medicine Strategy 2025–2034 further advanced this integration, emphasizing the necessity of evidence-based approaches to traditional, complementary, and integrative medicine within global health systems (1). Cultural heritage, deeply embedded in local practices and history, forms the bedrock of traditional medicine systems globally (1, 4). The WHO has increasingly recognized the link between cultural heritage and public health policies, aiming to integrate evidence-based TCIM, which is intrinsically linked to cultural heritage, into national health systems to achieve UHC (1, 2). Historically, the promotion of traditional medicine by the WHO, dating back to the late 1960s, emerged against a backdrop of decolonization and pan-Africanism, underscoring the political and cultural roots of these health systems. This historical context highlights how traditional medicine, as a significant aspect of cultural heritage, was championed as a means for self-determination and local health solutions (5).

As a vital part of traditional Chinese medicine, Mongolian medicine serves as a typical illustration of how national traditional medicines have evolved and integrated into national healthcare systems under the guidance of global policy trends and national supportive measures. In line with the WHO’s advocacy of recognizing the cultural relevance and clinical value of traditional medicines, China has incorporated Mongolian medicine—a treasure of its ethnic minority medical culture—into its national traditional medicine development framework, promoting its protection, inheritance, and modernization while upholding the global consensus on traditional medicine integration. Mongolian medicine represents the experience accumulated by the Mongolian people of China in their long-standing struggle against disease. Through extensive historical development as well as exchanges and integration with the medical cultures of fraternal ethnic groups in China, Mongolian medicine has gradually taken shape and evolved. Its theories, diagnostic methods, and treatments possess distinct ethnic and regional characteristics and have played a significant role in public health. Following the establishment of the People’s Republic of China, as supportive policies and regulations on traditional Chinese medicine (TCM) have been introduced continuously, Mongolian medicine entered a period of rapid modernization and development. It constitutes an important component of traditional Chinese medicine. The *Traditional Chinese Medicine Law of the People’s Republic of China*, promulgated in 2016, clearly defines traditional Chinese medicine as a collective term for medical systems developed through the long-term practices of both Han and ethnic minority groups (10). This situation profoundly reflects the Chinese nation’s systematic understanding of life, health, and disease prevention and treatment, highlighting the profound heritage and unique theoretical foundations of traditional Chinese medicine culture (11). Although the Law of the People’s Republic of China on Traditional Chinese Medicine incorporates Mongolian medicine into the framework of traditional Chinese medicine (TCM), Mongolian medicine faces public health challenges that differ significantly from those of Han Chinese medicine. First, heterogeneity of theoretical systems: Mongolian medicine is centered on the core theory of the “Three Roots and Seven Essentials” (Huyi, Xila, Badagan), emphasizing harmony between humans and nature. Its diagnostic methods (e.g., pulse diagnosis, urine diagnosis) and medication principles (e.g., cold–heat balance) resemble those of Han Chinese medicine in form but differ fundamentally in essence, requiring an independent standardization system and talent development pathway. Second, uniqueness of medication practice: Mongolian medicine widely uses processed mineral and animal drugs such as copper ash, silver ash, and mercury, which pose special technical challenges for quality control, toxicological evaluation, and clinical safety supervision. Third, uneven distribution of resources: Mongolian medicine resources are highly concentrated in border pastoral areas, making issues such as grassroots service accessibility, medical insurance coverage, and cross-regional circulation more prominent than for Han Chinese medicine. Fourth, oral transmission of inheritance: Extensive Mongolian medicine knowledge has been passed down through oral instruction and personal mentoring, placing it at risk of loss. Digital preservation, intellectual property protection, and cultural inheritance mechanisms urgently need to be established.

The *Opinions on Promoting the Inheritance and Innovative Development of Traditional Chinese Medicine* issued by the Central Committee of the Communist Party of China and the State Council in 2019 emphasized that traditional Chinese medicine is an important component of Chinese culture and called for strengthening the inheritance and innovative development of TCM culture (12). This document provides policy support for the cultural inheritance of and innovations in traditional Chinese medicine (including Mongolian medicine). The formulation and research of policies for the cultural inheritance of and innovations in traditional Chinese medicine (including Mongolian medicine) serve as crucial foundations for preserving the essence of TCM while fostering innovation. Given the relatively limited research in this field, this study analyzes national policy texts on the cultural inheritance of and innovations in traditional Chinese medicine (including Mongolian medicine) from 2016–2024 from the perspective of policy tools.

Policy tools are a series of operable intervention means, governance mechanisms, institutional arrangements, and action measures adopted by policy-making bodies to achieve established policy objectives (13, 14). They are the core intermediary and implementation carrier connecting policy objectives and policy results. Policy objectives are predefined, hierarchical, measurable results and value-oriented outcomes that public authorities (e.g., governments, international organizations and public sectors) aim to achieve through public intervention within a specific policy cycle (14, 15). They are the logical starting point, core orientation, and final evaluation benchmark of all policy actions. Policy objectives are the fundamental basis for tool selection, and policy tools are the core guarantee for goal achievement. In recent international health policy and public health governance research, policy tools were defined as all intervention measures adopted by governments and health authorities in the fields of health financing, service delivery, regulatory governance, and resource allocation to achieve health policy objectives. Policy objectives were further refined as strategic and phased outcomes anchored in the core areas of public health, medical security, health equity, and health system resilience (16–19). They are divided into two hierarchical levels in the valid literature, strategic (ultimate) objectives and intermediate (operational) objectives. Policy coherence depends on the hierarchical coordination of objectives and tools. Effective policies in complex health systems need to build a coherent logical chain of “strategic objectives – intermediate objectives – policy tools” to avoid the mismatch between objectives and tools. Balanced policy mix and multi-dimensional evaluation are the key to improving effectiveness. A single policy tool is difficult to achieve multi-dimensional health policy objectives, requiring a balanced policy combination, and a multi-dimensional evaluation framework to continuously calibrate the adaptability of objectives and tools. Dynamic adaptation of objectives and tools in an uncertain environment. Policy objectives need to maintain strategic stability, while policy tools need to be flexible and resilient (20).

While policy instrument analysis has emerged as a widely adopted analytical framework in scholarly inquiries into TCM policy, with a small but growing body of preliminary applications in ethnic medicine research, the existing literature suffers from three key and interrelated limitations that leave critical research gaps unaddressed. First, existing studies are marked by an overly generalized scope of inquiry and a lack of targeted focus. Most extant research either frames Mongolian medicine as a secondary, ancillary component of mainstream TCM in its analysis, or restricts its focus to a single category of policy instruments, most commonly supply-side interventions. To date, no study has systematically examined the holistic configuration of supply-, environment-, and demand-side policy instruments, with the inheritance and innovation of Mongolian medicine culture as its explicit core research focus. Second, the existing literature relies heavily on a one-dimensional analytical approach. The majority of relevant studies limit their analysis to descriptive frequency statistics of policy instruments, and fail to integrate policy instruments and their corresponding policy objectives into a unified, two-dimensional analytical framework. This methodological constraint means that existing research is unable to rigorously unpack the alignment, or critical misalignment, between deployed policy instruments and the core strategic objectives they are designed to achieve. Third, there is substantial homogeneity in the dominant research perspectives adopted in existing studies. Most textual analyses of ethnic medicine policy approach the topic from the lens of industrial development or clinical service provision, with very few studies conducting systematic, in-depth analysis from the dual perspective of cultural inheritance and innovation—a framing that uniquely integrates both the preservation of intangible cultural heritage and the modernized transformation of traditional medical systems. Against this backdrop, the present study aims to address these identified research gaps. Specifically, we construct a two-dimensional analytical framework linking policy instruments to core policy objectives, to systematically diagnose the structural imbalances in existing policies governing the inheritance and innovation of Mongolian medicine culture. In doing so, this study provides both a robust theoretical foundation and empirical evidence for the optimization of ethnic medicine policy in China.

A growing body of scholarship has applied policy tool analytical frameworks to traditional medicine and regional healthcare policy research, laying a robust methodological foundation for this study. In ethnic medicine research—the field most closely aligned with our core focus—this framework has been widely adopted for quantitative textual analysis of national and regional policy documents: existing studies have used multi-dimensional policy tool typologies to code Chinese ethnic medicine policies across central and local administrative tiers, revealing significant inter-tier differences in policy instrument selection preferences and widespread structural imbalances in instrument configuration (21, 22); yet these studies largely take the entire ethnic medicine industry as their analytical object, and have not yet conducted targeted, systematic analysis of policy instruments for the inheritance and innovation of Mongolian medicine culture, the core theme of this paper. In the broader field of TCM policy, this framework has become a mainstream analytical paradigm, with mature applications across sub-fields including TCM industrialization, health services, and medicine traceability systems, where related studies have consistently identified structural imbalances in policy instrument deployment and provided a critical reference for the design of our analytical dimensions (23–25). At the regional healthcare policy level, numerous studies have applied this framework to characterize local traditional medicine policy configuration, confirming that structural imbalances in policy instrument use are a common cross-regional phenomenon, while also highlighting the necessity of targeted regional analysis for geographically concentrated ethnic medicine systems such as Mongolian medicine, which informs our regional focus on Inner Mongolia (26).

Employing the three-dimensional policy instrument typology developed by Rothwell and Zegveld as its core analytical framework, which classifies policy interventions into supply-side, environment-side, and demand-side categories. This framework is selected for its near-perfect alignment with our core research focus: a systematic cross-sectional analysis of the structural configuration of policy instruments for the inheritance and innovation of Mongolian medicine culture, using policy texts spanning 2016 to 2024. Its key strength lies in its mature, operationalizable approach to converting qualitative policy texts into quantifiable coding units, enabling rigorous assessment of the distribution, internal composition, and target alignment of deployed policy instruments—an approach extensively validated in prior health policy and innovation policy scholarship. We do not adopt two prominent alternative public policy frameworks: the Advocacy Coalition Framework (ACF) and Policy Network Analysis (PNA). This exclusion is justified by a fundamental analytical mismatch: the ACF centers on explaining long-term dynamic policy change within policy subsystems, while PNA focuses on actor interactions and resource dependencies in the policymaking process, neither of which aligns with our static, text-based structural assessment of policy instrument configuration.

This study made explicit theoretical contributions: First, it extends the application scope of Rothwell and Zegveld’s policy tool classification theory to the field of traditional Chinese medicine (Mongolian medicine) cultural inheritance and innovation, enriching the theoretical application scenarios of policy tool theory in the traditional medicine and cultural heritage fields. Second, it constructs a two-dimensional analysis framework integrating policy tools and policy objectives, which fills the theoretical gap of systematic policy text analysis in the specific field of Mongolian medicine cultural inheritance and innovation, providing a theoretical framework and analytical paradigm for subsequent similar policy text studies in ethnic traditional medicine fields. Third, it complements the theoretical research on the matching relationship between policy tools and policy objectives in the field of traditional medicine cultural inheritance, providing theoretical support for the optimization of policy tool configuration and the balanced layout of policy objectives. Empirically, the configuration of policy tools is imbalanced structurally. The configuration pattern of policy tools that “prioritizes supply and neglects demand” reflects the inherent defects of the policy system; the overall layout of policy objectives is unbalanced, and the support is insufficient for basic policy objectives. Supply-type policy tools should be optimized, the proportion of demand-type policy tools should be increased, the linkage and complementarity among policy tools should be enhanced, the distribution of policy objectives should be balanced, support for basic policies should be strengthened to maximize their effectiveness, and the matching path between policy tools and objectives should be optimized.

## 2 Materials and methods

### 2.1 Materials

This study selected keywords such as “traditional Chinese medicine,” “Mongolian medicine,” “traditional Chinese medicine (Mongolian medicine),” and “cultural inheritance and innovation” to search for policy documents, including guidelines, notices, outlines, and plans related to the national cultural inheritance of and innovations in traditional Chinese medicine. Sources included relevant websites of the State Council, the Health Commission of Inner Mongolia Autonomous Region, the Medical Insurance Bureau, the Administration of Traditional Chinese Medicine, and databases such as the National Laws and Regulations Database and the PKULAW Database. The search period was 2016–2024. The selection criteria were as follows: [1] policies must be closely related to traditional Chinese medicine (Mongolian medicine), the cultural inheritance thereof, and innovations therein, and documents were excluded if they had repeated content or lacked substantive information (27); [2] issuing authorities were limited to the State Council and the Inner Mongolia Autonomous Region; and [3] document types included policy files such as plans, guidelines, notices, laws, and regulations that reflected the intentions of the national and Inner Mongolia Autonomous Region authorities. After retrieval and screening, 30 documents were included (Table 1). Notably, this study adopts a population-based study design rather than sampling, aiming to reveal the core structure of Mongolian medicine policies rather than making statistical inferences for all regions. Although the 30 samples are limited in number, they cover a complete time period and full policy levels, and thus can systematically reflect the structural characteristics of policies in this field.

**Table 1:** Selected policy documents on the cultural inheritance of and innovations in traditional Chinese medicine (Mongolian medicine).

Index	Policy name	Release Time (Year)	Issuing agency
3	Development Plan for Mongolian Medicine and Traditional Chinese Medicine Health Services in the Inner Mongolia Autonomous Region (2016–2020)	2016	General Office of the People’s Government of Inner Mongolia Autonomous Region
4	Implementation Plan for Promoting the Healthy Development of the Pharmaceutical Industry in the Inner Mongolia Autonomous Region	2017	General Office of the People’s Government of Inner Mongolia Autonomous Region
5	Action Plan for Revitalizing Mongolian Medicine in the Inner Mongolia Autonomous Region (2017–2025)	2018	General Office of the People’s Government of Inner Mongolia Autonomous Region
⋮	⋮	⋮	⋮
27	Notice on Promoting the Cultural Dissemination Campaign of Traditional Chinese Medicine (Mongolian Medicine)	2021	Health Commission of Inner Mongolia Autonomous Region
28	The 14th Five-Year Plan for Traditional Chinese Medicine (Mongolian Medicine) of the Inner Mongolia Autonomous Region	2022	Health Commission of Inner Mongolia Autonomous Region
29	Notice on Several Policy Measures to Promote the Characteristic Development of Traditional Chinese Medicine (Mongolian Medicine)	2022	General Office of the People’s Government of Inner Mongolia Autonomous Region
30	Implementation Plan for the “Healthy Inner Mongolia: Traditional Chinese Medicine (Mongolian Medicine) Culture Northern Border Tour”	2024	Health Commission of Inner Mongolia Autonomous Region

The selection of the 30 policy samples followed the principles of theme relevance, authority, representativeness, and common academic standards. The year 2016 was chosen as the starting point of the study, mainly because the Regulations on Traditional Chinese Medicine officially issued in that year clearly stipulated that the traditional Chinese medicine system includes traditional medicine of the Han nationality and minority medicine. This provided a legal basis for integrating Mongolian medicine into a unified policy framework and thus carried landmark significance. All samples were obtained from official and authoritative sources, including the central government, the People’s Government of Inner Mongolia Autonomous Region, the National Health Commission, and competent departments of traditional Chinese medicine. They focused on core areas such as cultural inheritance, innovative development, conservation, and utilization, which were highly consistent with the research theme. Non-normative documents such as temporary notices and simple letters were excluded, while formal policies with mandatory and guiding force were retained, including regulations, plans, opinions, and implementation plans. Meanwhile, the sample selection criteria of similar policy text analyses were referenced to ensure a complete time series, clear policy levels, and strong content representativeness. Finally, systematic coding of the 30 policies yielded 1,520 valid analysis units. The samples are scientific and reliable, and can comprehensively reflect the overall structure and evolutionary characteristics of policies for the cultural inheritance and innovation of traditional Chinese medicine (including Mongolian medicine) in China.

### 2.2 Methods

This study employs a content analysis to examine policy instruments. Using NVivo 15 software, selected policy documents were coded according to the structure “policy serial number—chapter/article—section—content,” resulting in 1,520 analysis units. On the basis of the classification of policy instruments, an analytical framework was further constructed to conduct a statistical analysis of the coding results. The coding scope for policy clauses related to service capacity building is formally defined to cover three interconnected core domains: clinical service capacity, which incorporates bed allocation and departmental capacity development of medical institutions, the geographic layout of the tiered medical service network, and the standardization of evidence-based diagnostic and therapeutic protocols; pharmaceutical management capacity, which involves regional accessibility of Mongolian medicines (with a specific focus on guaranteed stocking and availability in grassroots medical institutions), the establishment of a standardized rational medicine use monitoring system, and comprehensive pharmacovigilance capacity including the monitoring, reporting and emergency management of adverse reactions associated with Mongolian medicines; and quality improvement capacity, which encompasses standardized clinical pathway management, the systemization of clinical service specifications, and full-scope patient safety supervision and governance. Although this method can systematically reveal the structural characteristics of policy instruments, it still has certain limitations: First, text analysis only reflects the content design and value orientation of policy texts, and cannot directly measure the implementation effect and practical outcomes of policies. Second, the policy coding process relies on researchers’ subjective judgment and classification criteria, which may lead to certain interpretation biases. Third, limited by the sample scope, this study cannot cover all local and departmental policy documents. Finally, static text analysis is insufficient to fully demonstrate the dynamic evolution logic of policies and the multi-stakeholder interaction mechanism.

#### 2.2.1 Coding process and reliability test

In this study, the qualitative analysis software NVivo 15 was used to conduct systematic coding and content analysis on 30 policy texts. Coding strictly followed the principle of non-subdivisibility, with complete semantic units (clauses, paragraphs, etc.) as the minimum analytical unit. A single policy intention unit was recorded as one reference point, and units with multiple connotations were split into independent reference points until they could not be further subdivided (28). All codes were uniformly formatted as “policy serial number—chapter/article—section—content” to construct the analytical framework and count the coding results. Coding was completed by two researchers in the field of public health policy. Before formal coding, they received unified training to clarify the policy instrument classification framework and the operational definitions of nodes. To ensure objectivity and reproducibility, the two coders established independent projects in NVivo 15 and conducted coding back-to-back independently, and finally obtained 1,520 valid analysis units. Reliability testing was performed using the coding comparison function in NVivo (a reliability coefficient ≥ 0.80 was considered reliable) (29). The reliability test showed that the Cohen’s Kappa coefficient between the two coders was 0.9504, which was much higher than the critical standard of 0.80, indicating high coding consistency and credibility of the classification results. A small number of discrepant reference points were reviewed by a third-party expert to reach a consensus, and then merged to form the final coding library. Some coding results are shown in (Table 2).

**Table 2:** Content coding status of selected policy documents related to the cultural inheritance of and innovations in traditional Chinese medicine (Mongolian medicine).

Index	Policy name	Content analysis unit	Coding
5	Inner Mongolia Autonomous Region Action Plan for the Revitalization of Mongolian Medicine (2017–2025)	III. Key Tasks (2) Action to Enhance Mongolian Medicine Service Capacity 3. Strengthen the standardized provision and development of infrastructure, medical equipment, personnel allocation, and information systems, and establish regional Mongolian medicine diagnosis and treatment centers.	5–3–2-3
⋮	⋮	⋮	⋮
16	Implementation Opinions on Promoting the Inheritance and Innovative Development of Traditional Chinese Medicine (Mongolian Medicine)	V. Strengthening the Development of the Talent Team for Traditional Chinese Medicine (Mongolian Medicine) (3) Cultivating High-Level Talent in Traditional Chinese Medicine (Mongolian Medicine) 2. Thoroughly implement the national “Hundreds, Thousands, Tens of Thousands” Talent Project for the Inheritance of and Innovations in Traditional Chinese Medicine. Relying on national high-level talent-cultivation bases and projects as well as the autonomous region’s “Grassland Elite” project, vigorously cultivate and recruit a group of high-level talented individuals in traditional Chinese medicine (Mongolian medicine).	16–5–3-2
⋮	⋮	⋮	⋮
29	Notice on Several Policy Measures to Promote the Characteristic Development of Traditional Chinese Medicine (Mongolian Medicine)	III. Promoting the Development of the Traditional Chinese Medicine (Mongolian Medicine) Industry (2) Advancing the Marketization and Industrialization of Traditional Chinese Medicine (Mongolian Medicine) 1. Accelerate the translation and practical application of scientific and technological achievements in traditional Chinese medicine (Mongolian medicine), such as standard pharmaceutical preparations developed within medical institutions.	29–3–2-1

#### 2.2.2 Chi-Square goodness-of-fit test

To examine whether there are statistically significant structural differences in the distribution of supply-side, demand-side, and environmental policy instruments, this study adopted the chi-square goodness-of-fit test. The Pearson chi-square test is suitable for large-sample categorical problems without requiring strict normality assumptions, and can determine the significance by measuring the deviation between the observed and expected frequencies (30). In the testing procedure, the significance level was set at *α* = 0.05 (a universal standard in academia, indicating a confidence level of no less than 95%). The number of categories for the three types of policy instruments was *k* = 3. According to the formula for degrees of freedom df = k−1, the degrees of freedom were calculated as df = 2. From the chi-square distribution table, the critical value at α = 0.05 and df = 2 was $\chi_{0.05}^{2}(2)=5.991$ . The calculated chi-square statistic in this study was $\chi^{2}=125.84$ . By comparing this value with the critical value, since 125.84 was much larger than 5.991 (*p* < 0.001), the null hypothesis was rejected at the 0.05 significance level. This indicates that there are highly significant structural differences in the application distribution of the three types of policy instruments. The coding results are statistically reliable and can be used as a basis for subsequent research.

## 3 Construction of the two-dimensional analytical framework for policies related to the cultural inheritance of and innovations in traditional Chinese medicine (Mongolian medicine)

### 3.1 X dimension: policy instruments

This study adopts the policy instrument classification proposed by Rothwell and Zegveld (31) and categorizes policy instruments into three major types—supply-side, environment-side, and demand-side—which constitute the X dimension. The supply-side policy instruments primarily reflect the government’s direct intervention and resource investment in the process of cultural inheritance of and innovations in traditional Chinese medicine (Mongolian medicine) and support its development by providing essential elements such as talent, funding, and facilities (32). These instruments emphasize proactive government supply and contribute directly to the achievement of policy objectives. Environment-side policy instruments reflect the government’s efforts to promote the cultural inheritance of and innovations in traditional Chinese medicine (Mongolian medicine) through institutional environment development, including primarily institutional arrangements in areas such as planning, standards, regulations, supervision, and evaluation (33). These instruments create a favorable development environment and provide institutional guarantees for realizing policy goals. Demand-side policy instruments demonstrate the government’s approach to driving the cultural inheritance of and innovations in traditional Chinese medicine (Mongolian medicine) through market demand guidance and incentives, including aspects such as demonstration leadership, industrial support, and international cooperation (34). These instruments primarily mobilize social forces, stimulate market vitality, and generate synergistic policy effects. The specific classifications and interpretations of the policy instruments are detailed in Table 3.

**Table 3:** Categories and definitions of policy instruments for the cultural inheritance of and innovations in traditional Chinese medicine (Mongolian medicine).

Policy instrument type	Specific instruments	Definition
Supply-side	Talent development	Establishing a multilevel educational system and improving the talent cultivation mechanism.
	Information support	Establishing knowledge bases, digital resources, and dissemination platforms to deliver information services.
	Document collection and preservation.	Conducting surveys, collation, preservation, and digitalization of ancient texts.
	Service capacity building	Enhancing the standard of medical services, and improving service networks and capacity development.
	Infrastructure development for institutions	Developing facilities such as cultural exhibition venues, medical institutions, and educational bases.
Environment-side	Multiparty collaboration	Establishing interdepartmental collaboration mechanisms; promoting medical-care-education-research collaboration; advancing industry-academia-research integration.
	Policies and regulations	Improving the legal and regulatory framework; formulating specialized policies; establishing standards and norms.
	Incentive mechanisms	Establishing talent incentive systems; improving assessment, evaluation, and distribution mechanisms.
	Supervision and assessment	Establishing a quality supervision system, conducting performance evaluations and dynamic monitoring.
	Goal planning	Formulating development plans, defining strategic objectives, and implementing project management.
Demand-side	Industrial promotion and support	Supporting industrial development, fostering distinctive industries, and promoting transformation and upgrading.
	International exchanges and cooperation	Conducting cultural exchanges, advancing technological collaboration, and promoting educational and research cooperation.
	Policy support	Providing policy incentives, optimizing the development environment, and improving support measures.
	Demonstrative and leading role	Establishing demonstration bases, creating distinctive brands, and cultivating exemplary cases.

### 3.2 Y dimension: policy objective dimension

Policy objectives represent the anticipated outcomes of policy formulation and implementation and define the purpose of policy instruments. Therefore, the Y dimension in this study is used to analyze the policy objectives.

On the basis of a systematic review of laws and regulations such as the Traditional Chinese Medicine Law of the People’s Republic of China, the Traditional Chinese Medicine Regulations of the Inner Mongolia Autonomous Region, and the Mongolian Medicine Regulations of the Inner Mongolia Autonomous Region as well as policy documents including the “14th Five-Year Plan” for the Development of Traditional Chinese Medicine and the “14th Five-Year Plan” for the Development of Traditional Chinese Medicine (Mongolian Medicine) in the Inner Mongolia Autonomous Region, this paper develops an analytical dimension for the policy objectives related to traditional Chinese medicine (Mongolian medicine) cultural inheritance and innovation. The categorization of policy objectives fully considers the regional and ethnic characteristics of traditional Chinese medicine (Mongolian medicine) development, encompassing aspects such as talent, culture, theory, scientific research, standards, services, industry, and international cooperation. The classifications and descriptions of the specific objective dimensions are detailed in Table 4.

**Table 4:** Categories and definitions of policy objectives.

Policy objectives	Definitions
Strengthen the talent team development of traditional Chinese medicine (Mongolian medicine).	Cultivate and introduce professionals in traditional Chinese medicine (Mongolian medicine), build a multilevel talent system, and enhance the overall competency of the talent team.
Enhance the institutional development for traditional Chinese medicine (Mongolian medicine) culture.	Improve the layout of institutions dedicated to the cultural inheritance of and innovations in traditional Chinese medicine (Mongolian medicine), and upgrade cultural dissemination and service capabilities.
Strengthen the research on ancient literature collation and theoretical innovation in traditional Chinese medicine (Mongolian medicine).	Advance the collation and preservation of ancient texts, deepen theoretical research, and promote the innovative development of traditional theories.
Strengthen the cultural inheritance and dissemination of traditional Chinese medicine (Mongolian medicine).	Intensify publicity efforts for traditional Chinese medicine (Mongolian medicine) culture, promote its integration into public life, and develop modern creative products and cultural services related to traditional Chinese medicine (Mongolian medicine).
Advance standardization projects for traditional Chinese medicine (Mongolian medicine).	Establish and improve the standard system, enhance quality standards, and advance standardization efforts.
Enhance the service capacity of traditional Chinese medicine (Mongolian medicine).	Improve the standard of medical services, optimize the service network, and strengthen service delivery capabilities.
Enhance the scientific research capability and achievements translation of traditional Chinese medicine (Mongolian medicine).	Strengthen technological innovation capacity, promote the translation of research outcomes into clinical and industrial applications, and improve innovation efficiency.
Deepen international exchange and cooperation in traditional Chinese medicine (Mongolian medicine).	Promote international exchanges, conduct collaborative research, and enhance international influence.

### 3.3 Two-dimensional analytical framework

**Figure 1:**
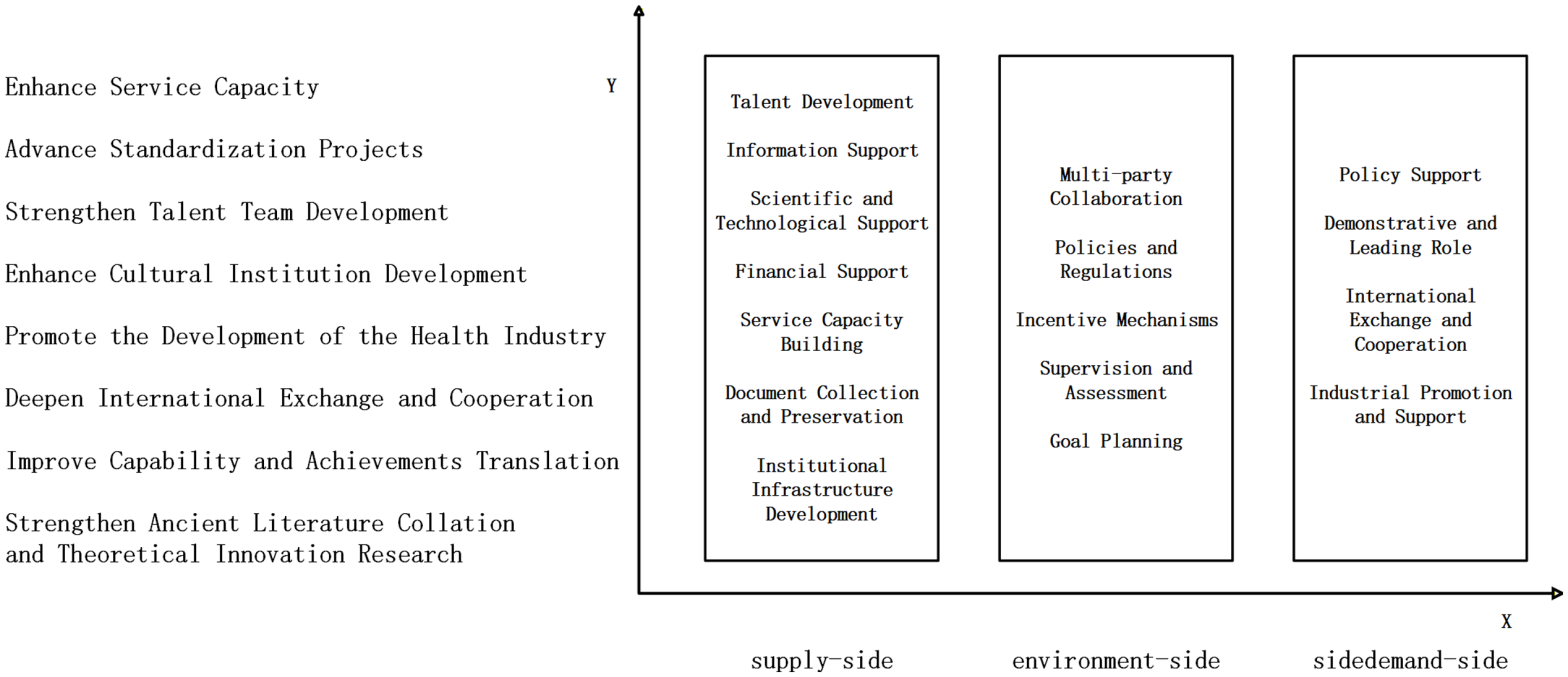
Two-dimensional analytical framework for the cultural inheritance of and innovations in traditional Chinese medicine (Mongolian medicine).

On the basis of the comprehensive analysis of the policy instrument and objective dimensions discussed above, this paper constructs a two-dimensional analytical framework for policies on the cultural inheritance of and innovations in traditional Chinese medicine (Mongolian medicine), as illustrated in Figure 1.

## 4 Results

### 4.1 X dimension: analysis results of policy instruments

After 30 policy documents were selected and coded, 1,520 valid analysis units resulted. The distribution of policy instruments is shown in Table 5.

**Table 5:** Distribution of basic policy instruments.

Policy instrument type	Specific instruments	Frequency	Proportion (%)
Supply-side	Talent development	96	6.32
	Information support	145	9.54
	Document collection and preservation	133	8.75
	Service capacity building	133	8.75
	Infrastructure development for institutions	24	1.58
Environment-side	Multiparty collaboration	52	3.42
	Policies and regulations	39	2.56
	Incentive mechanisms	50	3.29
	Supervision and assessment	23	1.51
	Goal planning	99	6.51
Demand-side	Industrial promotion and support	142	9.34
	International exchanges and cooperation	283	18.63
	Policy support	112	7.37
	Demonstrative and leading role	81	5.33

Overall, the configuration of policy instruments presents a distinct pattern of supply-led, environment-supported, and demand-supplemented. Supply-side instruments account for the largest proportion (40.92%), indicating a strong path dependence in which the government promotes the development of Mongolian medicine through direct resource input in personnel, facilities, and technology. This reflects policymakers’ preference for tangible, visible infrastructure construction. Demand-side instruments account for only 19.80%, implying insufficient mobilization of market and social forces, and a weak response of the policy system to external incentive mechanisms and consumption-side drivers, which may restrict the marketization process of the Mongolian medicine industry.

Within supply-side instruments, resource allocation is biased toward hardware over software and construction over support. Infrastructure construction (9.54%), scientific and technological support (8.75%), and talent training (8.75%) constitute the three core pillars, embodying a policy logic of “infrastructure first, innovation-driven, and talent-guaranteed”. However, literature collection and preservation account for only 1.58%, and financial support for only 2.56%. This structure suggests that the policy system favors investments with short-term visible outcomes, while paying insufficient attention to culturally foundational work such as ancient book protection and rescue of oral historical materials and long-term sustainable guarantees such as stable financial input mechanisms, which may weaken the sustainability of Mongolian medicine inheritance.

Within environment-side instruments, policies and regulations (18.63%) are absolutely dominant, highlighting the strong role of institutional governance. The relatively high proportions of goal planning (9.34%) and supervision and evaluation (6.51%) reflect a strategic orientation and process control tendency in policy implementation. Nevertheless, multi-stakeholder collaboration (3.29%) and incentive mechanisms (1.51%) account for relatively low shares, indicating that cross-sectoral coordination and endogenous motivation remain policy shortcomings, which may lead to a “fragmentation” dilemma in policy implementation.

Although demand-side instruments account for the lowest overall proportion, they present a reasonable internal gradient structure. Industrial promotion (7.37%) serves as the main engine, forming a synergistic chain with international cooperation (5.33%) and policy support (4.80%). However, demonstration and leadership account for only 2.30%, suggesting that the policy diffusion mechanism of “promoting the whole through typical cases” has not been effectively established, and the ability to replicate and promote mature experience needs strengthening. This configuration implies that current demand-side policies are insufficient to form strong market-driven forces for the development of Mongolian medicine.

### 4.2 Analysis results of the Y dimension: policy objectives

The policies for the cultural inheritance of and innovations in traditional Chinese medicine (Mongolian medicine) contain 160 coding units pertaining to development objectives. The data analysis reveals that talent team development occupies a strategic core position, accounting for the greatest proportion at 25.63% of the total. Service capacity (15.00%), health industry development (14.38%), and scientific research capability with achievement translation (14.38%) constitute the second tier of key objectives. Moreover, international exchange and cooperation (8.75%), cultural institution development (8.75%), ancient literature collation and theoretical innovation research (6.88%), and standardization projects (6.25%) form the foundational supporting layer of the policy objectives. The distribution is detailed in Table 6.

**Table 6:** Distribution of policy objectives.

Index	Policy objectives	Frequency	Proportion (%)
1	Standardization projects	10	6.25
2	Service capacity	24	15
3	Ancient literature collation and theoretical innovation research	11	6.88
4	International exchange and cooperation	14	8.75
5	Health industry development	23	14.38
6	Scientific research capability and achievements translation	23	14.38
7	Talent team development	41	25.63
8	Cultural institution development	14	8.75
Total		160	100.00

### 4.3 Results of the two-dimensional cross-analysis between policy instruments and policy objectives

The two-dimensional cross-analysis of policy instruments and policy objectives reveals a complex and multilayered policy mechanism, as shown in Table 7.

**Table 7:** Two-dimensional cross-analysis results of “Policy Instruments–Policy Objectives” [*n* (%)].

Policy objectives	1. supply-side	2. environment-side	3. demand-side	Total
	117 (46.06)	81 (31.89)	56 (22.05)	254 (100)
1. Talent team development	35 (61.40)	19 (33.33)	3 (5.26)	57 (100)
2. Health industry development	17 (43.59)	9 (23.08)	13 (33.33)	39 (100)
3. Ancient literature collation and theoretical innovation research	11 (36.67)	13 (43.33)	6 (20.00)	30 (100)
4. International exchange and cooperation	5 (20.83)	5 (20.83)	14 (58.33)	24 (100)
5. Cultural institution development	10 (33.33)	10 (33.33)	10 (33.33)	30 (100)
6. Service capacity	13 (44.83)	11 (37.93)	5 (17.24)	29 (100)
7. Standardization projects	5 (50.00)	3 (30.00)	2 (20.00)	10 (100)
8. Scientific research capability and achievements translation	21 (60.00)	11 (31.43)	3 (8.57)	35 (100)

Percentages in parentheses indicate row percentages.

Among the objectives for talent team development, supply-side policy instruments account for the greatest proportion (35.00). In terms of the objectives for scientific research capability and achievement translation, supply-side policy instruments rank second (21.00). The supply-side policy instruments also constitute relatively high proportions of the objectives for health industry development and service capacity (17.00 and 13.00, respectively). With respect to talent team–development objectives, environmental policy instruments constitute the greatest proportion (19.00). With respect to the objectives of ancient literature collation and theoretical innovation research, environmental policy instruments account for a relatively high proportion (13.00). In terms of the objectives for international exchange and cooperation and health industry development, demand-side policy instruments occupy relatively high proportions (14.00 and 13.00, respectively). Demand-side policy instruments also account for a notable proportion of the objectives for cultural institution development (10.00).

**Figure 2:**
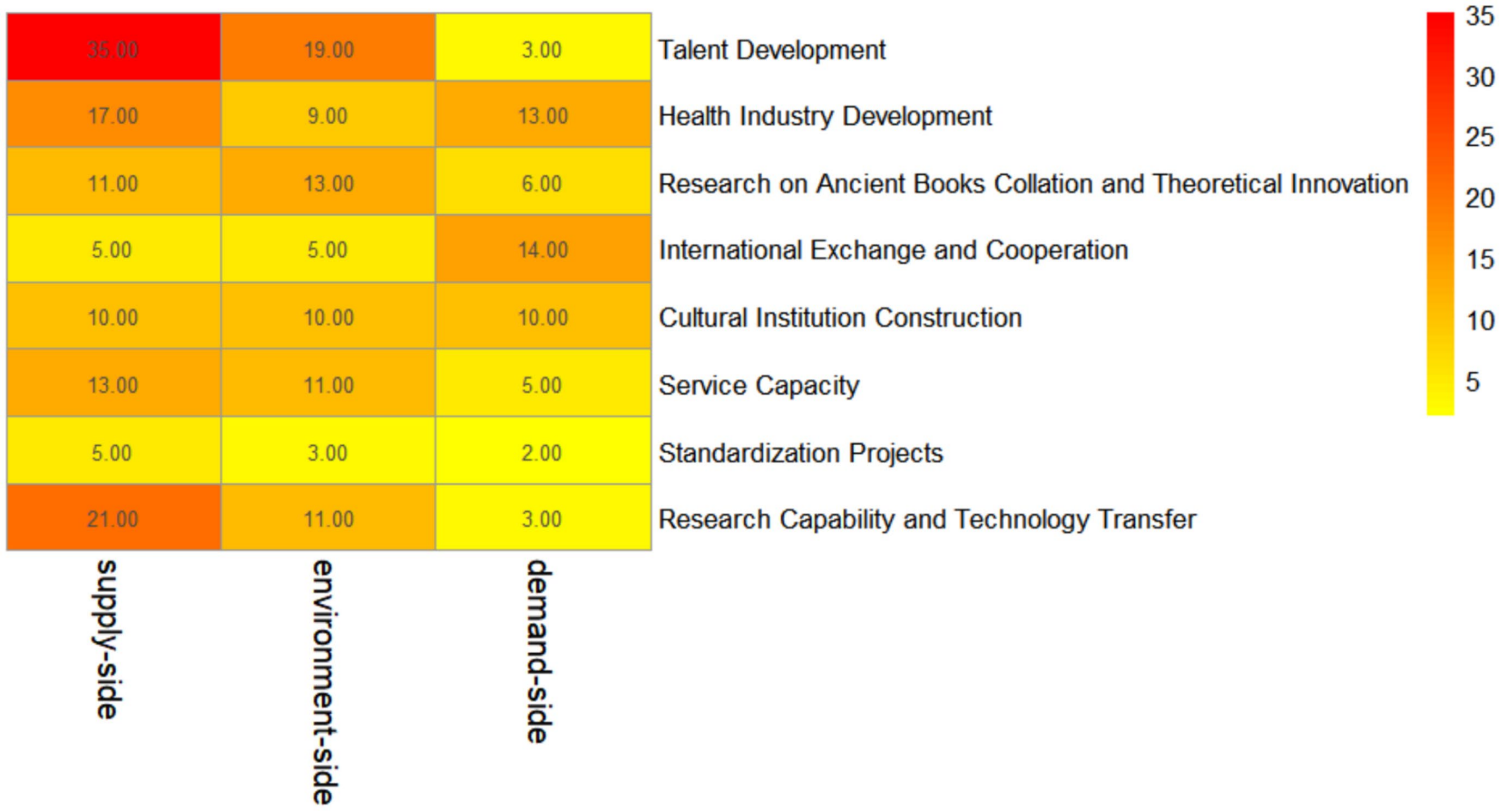
Heatmap representation of policy instruments.

As illustrated by the intersectional intensity shown in the heatmap, there are significant variations in the correlation strength between policy instruments and policy objectives, as demonstrated in Figure 2. This analysis reveals the co-occurrence patterns and correlation strengths between specific policy instrument types and policy objectives.

Overall, the cross-distribution between policy instruments and policy objectives exhibits a hierarchical characteristic of “supply-led, environment-supported, and demand-supplemented”. The supply-side policy instruments maintain a high correlation intensity across most policy objectives, reflecting their dominant role in the policy system. Environmental policy instruments demonstrate a relatively balanced supportive function, whereas demand-side policy instruments, although strongly correlated with certain specific objectives, exhibit an overall lower correlation intensity. This analysis delineates the distinct co-occurrence patterns between core policy objectives and three categories of policy instruments (supply-side, environmental, and demand-side), demonstrating highly differentiated strategic reliance across various policy priorities. Talent Team Development presents the strongest co-occurrence with supply-side policy instruments, which account for 35.00% of instrument usage for this goal, followed by a notable 19.00% co-occurrence with environmental instruments and a mere 3.00% with demand-side ones, highlighting a heavy dependence on direct government provision and support for talent cultivation. For Scientific Research Capability and Achievement Translation, supply-side instruments rank second in co-occurrence at 21.00%, underscoring the substantial role of direct resource allocation in advancing research and innovation. Health Industry Development features relatively high co-occurrence with both supply-side (17.00%) and demand-side (13.00%) instruments, reflecting a mixed strategy that combines direct government support and market-oriented incentives. International Exchange and Cooperation shows a prominent 14.00% co-occurrence with demand-side instruments, indicating that market-driven guidance and incentives are central to facilitating global engagement, while supply-side and environmental instruments each make a minor 5.00% contribution. For Ancient Literature Collation and Theoretical Innovation Research, environmental instruments account for a relatively high 13.00% of co-occurrence, signaling that institutional arrangements, regulations, and planning are critical to underpinning these foundational activities, with supply-side instruments also contributing 11.00%. Cultural Institution Development displays a balanced co-occurrence across all three instrument categories, each representing 10.00% of the support, pointing to an integrated approach for cultural infrastructure development. Finally, Standardization Projects exhibit the weakest overall co-occurrence with policy instruments, with the highest share from supply-side instruments at only 5.00%, trailed by environmental (3.00%) and demand-side (2.00%) instruments, suggesting that policy support for standardization efforts receives notably less emphasis compared to other policy objectives.

### 4.4 Dynamic evolution analysis

From 2016 to 2024, the policy instruments for the cultural inheritance and innovation of traditional Chinese medicine (including Mongolian medicine) in China have undergone a dynamic development process: initial layout – accelerated promotion – deepening and improvement. The three types of policy instruments (supply-side, environmental-side, and demand-side) have generally shown a pattern of “environmental-side dominance and support, steady optimization of supply-side, and fluctuating improvement of demand-side”.

As the core support for cultural inheritance and innovation, supply-side policy instruments accounted for 29.33–44.78% over the 9 years, demonstrating the evolutionary characteristics of steady optimization and phased focus. From 2016 to 2018, the focus was on infrastructure construction and basic talent training, laying a solid foundation for cultural inheritance. From 2019 to 2021, the emphasis shifted to talent quality improvement and scientific and technological support, strengthening connotation-oriented supply. From 2022 to 2024, priority was given to collaborative supply and achievement transformation to adapt to the needs of high-quality development. However, obvious deficiencies still exist in such instruments. The sub-tool of traditional Chinese medicine literature collation and protection has long maintained a low proportion. The collation and excavation of ancient books and folk prescriptions of Mongolian medicine are insufficient, failing to fully exert the inheritance value of traditional literature. Meanwhile, financial support is weak, with scattered special funds and inadequate support for projects on the inheritance and innovation of Mongolian medicine culture. It is difficult to meet the sustainable needs of talent training, scientific and technological research and development, which restricts the further improvement of supply quality.

Environmental policy instruments have maintained a dominant position throughout the period, accounting for 34.39–48.67%, and showed an evolutionary trend of “guidance – regulation – coordination”. From 2016 to 2017, the focus was on goal planning and policy guidance to clarify the development direction. From 2018 to 2021, emphasis was placed on improving regulations, incentives, and supervision to standardize the development path. From 2022 to 2024, efforts focused on collaborative empowerment and long-term guarantee to build a sound policy ecosystem. However, the application of sub-instruments is uneven. The incentive mechanism sub-instrument is insufficient: the recognition and rewards for talents and high-quality projects in the inheritance and innovation of Mongolian medicine culture are inadequate, which fails to fully stimulate social participation. The multi-stakeholder collaboration sub-instrument accounts for a relatively low proportion. The cross-departmental and cross-regional collaborative promotion mechanism is imperfect, and the linkage among the government, medical institutions, enterprises, and research institutes is insufficient, resulting in a failure to form joint forces to promote cultural inheritance and innovation. Meanwhile, policy texts fail to consider communication and coordination among multiple stakeholders, which may easily lead to cognitive bias and implementation bias (35).

As the driving force for the market and society, demand-side policy instruments maintained a share of 15.00–25.00% over the 9 years, showing an evolutionary trend of fluctuating improvement and gradual enhancement. From 2016 to 2018, the focus was on basic policy support and preliminary industrial promotion to cultivate market demand. From 2019 to 2021, efforts shifted to optimizing demand guidance and popularizing pilot experience. From 2022 to 2024, emphasis was placed on expanding international exchanges and industrial integration to strengthen demand-driven development. However, such instruments have the most prominent shortcomings. The sub-instrument of demonstration and leading role accounted for a relatively low proportion. The number of pilot demonstration projects was insufficient with limited coverage, making it difficult to effectively promote mature experience. Support for industrial promotion was inadequate. The marketization level of Mongolian medicine cultural products remained low, and the industrial chain was incomplete, failing to fully unleash market-driven potential. Only the sub-instrument of policy support gradually increased its proportion, providing a certain guarantee for demand-side development. Nevertheless, it still cannot compensate for the deficiencies of other sub-instruments overall, and the problem of imbalance between supply and demand has not been fundamentally resolved.

### 4.5 Causal and institutional interpretation of observed distributions

Predominance of supply-side instruments and underutilization of demand-side instruments: The “prioritizes supply and neglects demand” pattern reflects inherent defects in the policy system. This indicates a top-down approach where the government primarily focuses on providing resources and infrastructure (supply-side) rather than stimulating market demand or engaging social forces (demand-side). This imbalance could be attributed to the institutional framework where government agencies are more accustomed to direct intervention and resource allocation. The emphasis on institutional infrastructure development, scientific and technological support, and talent cultivation within supply-side instruments points to a state-led development model. The lower proportions for demand-side instruments like demonstrative and leading roles, international exchange and cooperation, and policy support suggest a less developed institutional capacity or willingness to leverage market mechanisms and external partnerships.

Imbalance within supply-side instruments: While institutional infrastructure development, scientific and technological support, and talent cultivation form the core support, the low proportions of document collection and preservation, information support, and financial support indicate potential gaps. This could be due to a focus on tangible assets and human capital over less visible but equally crucial foundational elements. The strong emphasis on institutional infrastructure and talent might stem from a policy-making culture that prioritizes visible achievements and direct control. The under-resourcing of document collection and preservation, for instance, might reflect a lack of institutional recognition for the long-term strategic value of cultural heritage documentation compared to immediate development goals.

Imbalance within Environmental Instruments: The high proportion of policies and regulations highlights a reliance on institutionalized governance. However, the lower proportions for multiparty collaboration and incentive mechanisms suggest a less adaptive and flexible governance approach. This indicates an institutional preference for formal rules and regulations to guide development, possibly due to a desire for control and standardization. The limited use of incentive mechanisms and collaboration might point to institutional barriers in fostering broader stakeholder engagement or a lack of trust in market-driven incentives.

Imbalanced Distribution of Policy Objectives: The strategic core position of talent team development reflects a “people-oriented” development philosophy, highlighting the foundational and decisive role of talent. The lower proportions for ancient literature collation and theoretical innovation research, and standardization projects, despite their strategic value, suggest that these foundational areas might be perceived as less urgent or impactful in the short term. The prioritization of talent development, service capacity, health industry development, and scientific research capability aligns with institutional goals of rapid modernization and economic growth. The relative neglect of foundational aspects like ancient literature and standardization could be a consequence of policy-makers focusing on immediate, measurable outcomes rather than long-term, systemic strengthening. This might also reflect a lack of dedicated institutional structures or funding mechanisms for these specific foundational areas.

Imbalance in the Use of Policy Instruments within Policy Objectives: The strongest correlation between supply-side instruments and talent team development, while logical, also indicates insufficient support for other objectives. For example, the weak correlation of supply-side instruments with international exchange and cooperation, and standardization projects, suggests that the current supply-side approach is not effectively addressing these areas. Similarly, the “balanced but weak” characteristic of environmental policy instruments across objectives indicates that while they provide general support, they lack the intensity to drive specific outcomes effectively. The evident insufficiency of demand-side policy instruments across various objectives means that market mechanisms are not playing a decisive role in resource allocation. This misalignment points to a lack of integrated policy design. Different policy instruments might be designed and implemented in silos, without sufficient consideration for their synergistic effects on various objectives. The institutional structures might not be conducive to cross-instrument coordination, leading to fragmented efforts and suboptimal outcomes, particularly for objectives that require a blend of supply, environmental, and demand-side interventions. The weak role of demand-side instruments further highlights an institutional reluctance or inability to fully embrace market-driven approaches.

### 4.6 Global paradigm contrasts and policy implications

China’s traditional and complementary medicine (T&CM) strategy is a definitive example of the state-led model, marked by robust top-down government investment in T&CM infrastructure, talent cultivation, and regulatory system building—an approach typical of nations where traditional medicine is deeply embedded in national identity, universal healthcare frameworks, and the state’s central role in steering socioeconomic development. This strong state-centric pattern is similarly evident in peer jurisdictions: India allocates substantial government funding to research councils, educational institutions, and clinical facilities for its AYUSH (Ayurveda, Yoga & Naturopathy, Unani, Siddha, and Homoeopathy) systems (36), paired with comprehensive regulatory frameworks for practitioners and pharmaceutical manufacturing, while South Korea adopts a comparable state-led approach for its Korean Medicine system, with both countries also prioritizing talent development and public service provision within their traditional medicine ecosystems. In sharp contrast, the policy landscape for Complementary and Alternative Medicine (CAM) across most Western countries (including EU member states, the United States, and Canada) is predominantly market-driven and regulation-focused: government intervention here is far less centered on direct supply-side resourcing, and instead focuses primarily on consumer protection, practitioner regulation via environmental-side policy tools, and targeted funding for research evaluating CAM efficacy and safety (37); where demand-side policy instruments are deployed, they are typically limited to insurance coverage for specific CAM therapies rather than the broad, cross-cutting industrial promotion seen in state-led models, meaning the “supply-led, demand-neglecting” critique highlighted in the China-focused study is largely inapplicable in contexts where market forces are the primary driver of demand and the government’s core role is supervisory rather than directive.

Empirical analyses of TCM policy in China show that its strategic priorities center on talent development, clinical capacity building, health industry expansion, scientific innovation, and cultural inheritance, with widely documented structural limitations stemming from insufficient policy focus on foundational research and system-wide standardization. This priority structure contrasts sharply with that of Japan’s Kampo medicine, a global benchmark for successful Traditional, Complementary and Alternative Medicine (TCAM) integration into mainstream biomedical care. Existing scholarship confirms that Japan’s Kampo policy prioritizes rigorous scientific validation of efficacy and safety, standardized medical education for licensed practitioners, and full-lifecycle standardization of production, prescription and quality control—measures instrumental to Kampo’s formal clinical acceptance and national health insurance coverage, directly opposing the limited standardization focus seen in Chinese TCM policy. A distinct alternative paradigm is found in Indigenous traditional medicine policy across Canada, Australia and New Zealand, where priorities center on cultural preservation, intergenerational knowledge transmission and community self-determination, rather than commercialization or biomedical integration. Policy tools in these contexts focus on legal recognition, intellectual property protection and community-led initiative funding, aligning with the supply-side and environmental-side policy typologies outlined in prior analysis.

The Chinese study’s results align with a broader understanding of state-led development models for traditional medicine, particularly in Asian countries with rich T&CM heritage. The strong emphasis on supply-side and environmental tools, and the prioritization of talent and service capacity, are common features. However, the identified “supply-led, neglects demand” imbalance and the under-prioritization of foundational elements like standardization and theoretical innovation highlight specific areas where China’s policy system could learn from or be contrasted with other models. For instance, Japan’s success with Kampo suggests that robust standardization and scientific validation are critical for deeper integration, while Western CAM policies, though less interventionist, often prioritize consumer safety through strong environmental regulations. The study provides a valuable case study for understanding the complexities of governing traditional medicine in a context of rapid modernization and cultural preservation, offering insights that can inform and be informed by comparative research globally.

### 4.7 The implications for public policy design

This empirical analysis of policy texts governing the cultural inheritance and innovation of Traditional Chinese Medicine (Mongolian Medicine) in China provides robust, evidence-based implications for traditional medicine public policy design, rooted in the identification of three fundamental structural flaws in the existing framework: pronounced imbalance in policy instrument allocation, skewed distribution of strategic policy objectives, and substantial misalignment between policy instruments and their targeted priority goals. The study documents a dominant “supply-led, environment-supported, demand-supplemented” policy configuration, marked by overreliance on supply-side instruments and marked underutilization of demand-side tools, alongside insufficient policy attention and resource allocation to foundational objectives including ancient literature collation, theoretical innovation, and standardization, despite appropriate prioritization of talent development. It further identifies critical gaps within instrument categories, overreliance on rigid regulation, and weak instrument-objective alignment, alongside the failure to fully leverage market mechanisms and social forces in resource allocation. Accordingly, core policy implications include the imperative to transition to a balanced, synergistic policy tool portfolio, strategically re-evaluate objective prioritization and cross-objective resource allocation, enhance policy precision and adaptability, establish a dynamic instrument-objective matching mechanism, and innovate market-oriented support modalities. Collectively, these findings mandate a paradigm shift from a predominantly supply-driven model to a comprehensive, integrated policy framework that aligns cross-category policy instruments and fosters robust cross-cutting synergy across the entire traditional medicine policy ecosystem.

## 5 Discussion

### 5.1 Structural imbalance in policy instrument allocation

The systematic analysis of policy instrument allocation clearly shows that the current policy framework for the cultural inheritance of and innovations in traditional Chinese medicine (Mongolian medicine) exhibits significant structural imbalances. The supply-side and environmental policy instruments occur frequently, whereas demand-side policy instruments are relatively scarce.

Although supply-side policy instruments are employed extensively, their internal structure is imbalanced. Elements such as institutional infrastructure development, scientific and technological support, and talent cultivation are supplied at relatively high rates, forming core support on the supply side. Service capacity building is relatively well supported. However, information support remains insufficient, and critical areas such as document collection and preservation as well as financial support occupy notably low proportions of policy instrument allocation. Such structural contradictions may hinder the balanced development of the industry.

Environmental policy instruments are also utilized at a high frequency, but their internal structure similarly shows significant imbalances. Policies and regulations occupy the greatest proportion, highlighting the dominant role of institutionalized governance. However, there were prominent shortcomings such as ambiguous division of powers and responsibilities and unreasonable allocation of fiscal resources (38). The relatively significant proportions of goal planning as well as supervision and assessment reflect the strategic orientation and process control tendencies in policy implementation. The lower proportions allocated to multiparty collaboration and incentive mechanisms indicate room for improvement in adaptive governance and motivational measures within the policy system.

Demand-side policy instruments are relatively underutilized. Industrial promotion and support account for a greater proportion of the total, serving as the primary means of stimulating demand. However, the lower proportions allocated to international exchange and cooperation as well as policy support weaken the ability of the policy system to stimulate market demand and promote internationalization. The minimal allocation to demonstrative and leading roles may hinder the effective functioning of exemplary models and leadership-driven initiatives.

From a health economics perspective, the mere 2.56% share of financial support instruments in China’s Mongolian medicine policy framework constitutes a critical structural constraint to the sector’s cultural inheritance, innovation and sustainable development. While regulatory, planning and infrastructure instruments establish the necessary institutional framework, they cannot substitute for the foundational enabling role of sustained fiscal support. This funding shortfall disproportionately harms resource-constrained grassroots Mongolian medicine institutions, undermining their operational stability, talent retention, service capacity, research innovation and core cultural inheritance work, while creating a misalignment between stated policy priorities and committed fiscal resources, alongside tangible over-commercialization risks. Over the long term, this imbalanced policy architecture—overreliance on regulatory environmental instruments paired with insufficient supply-side fiscal support—erodes the sector’s developmental resilience. Left unaddressed, this critical fiscal gap will ultimately undermine the core policy objectives of Mongolian medicine’s cultural inheritance and innovation.

Taken together, these findings expose deep-seated structural imbalances across the full policy instrument typology, rather than being isolated to the supply-side dimension. Although this pattern of policy instrument utilization provides strong support for the industry’s development in the short term, from a long-term perspective, the policy instrument portfolio requires further optimization. In particular, demand-side policies must be strengthened to achieve development outcomes that are more balanced and sustainable development (39, 40).

### 5.2 Imbalanced distribution of policy objectives

The analysis of the policy objectives dimension reveals significant imbalances in the overall layout of the target system. Talent team development accounts for the greatest proportion of this development, with its core position being established. Service capacity, health industry development, and scientific research capability with achievement translation form the second tier of key objectives. International exchange and cooperation, along with cultural institution development, account for relatively lower proportions. The lowest proportions are allocated to ancient literature collation and theoretical innovation research as well as standardization projects.

This hierarchical distribution of policy objectives reflects the strategic logic and priorities of policy-makers. Placing talent team development at the forefront embodies a “people-oriented” development philosophy (41), highlighting the foundational and decisive role of talent in the cultural inheritance of and innovations in traditional Chinese medicine (Mongolian medicine). The high proportion allocated to service capacity indicates that policy-makers emphasize enhancing practical capabilities and service provision, a focus that interacts positively with the goal of health industry development, and together such policies form the core driving force for industrial growth (42).

The prominence of objectives related to scientific research capability and achievement translation reflects the policy system’s strong emphasis on technological innovation and practical application. This orientation helps enhance the innovative vitality and development potential of traditional Chinese medicine (Mongolian medicine) (43). International exchange and cooperation, in addition to cultural institution development, are positioned as equally important objectives, demonstrating a balanced approach to international engagement and infrastructure development. Such an allocation supports the intrinsic and extrinsic expansion of cultural inheritance and innovation.

Although ancient literature collation and theoretical innovation research account for a relatively low proportion of the total, their role as crucial carriers of traditional culture and foundational support for theoretical innovation grants them irreplaceable strategic value within the policy objective system. Similarly, standardization projects, despite occupying the lowest proportion among policy objectives, serve as key drivers for promoting industry standardization and quality enhancement, clearly demonstrating their importance. The mere 6.25% share of standardization-focused policy instruments represents a severe systemic gap that forms a fundamental barrier to the marketization of Mongolian medicine. From a pharmaceutical regulatory perspective, the clinical translation of in-hospital preparations is bound by a statutory “standards-first” mandate: under the Drug Administration Law of the People’s Republic of China and Measures for the Administration of Registration of Medical Institution Preparations, in-hospital preparations can only obtain filing or registration approval and subsequent inclusion in the medical insurance payment system if they meet comprehensive quality standards, including clear specifications for medicinal material origin, processing protocols, content determination, and stability testing. The chronic under-provision of standardization policy instruments has created three interlocked translation barriers: first, blocked market access, as numerous traditional Mongolian medicine formulas fail to secure preparation filing approval due to the lack of unified quality standards, leaving them in a long-term state of “validated prescriptions without marketable medicinal products”; second, restricted insurance coverage, as non-standardized in-hospital preparations are ineligible for the medical insurance reimbursement catalog, driving up patient costs and suppressing clinical demand; and third, stagnant cross-regional circulation, as the absence of standardized benchmarks disqualifies Mongolian medicine preparations from cross-provincial dispensing approval, permanently confining their market scope to in-hospital use only. This critical standardization deficit has trapped Mongolian medicine in a paradox of “rich historical inheritance without modern translation, proven clinical efficacy without formal market access”, emerging as the core institutional bottleneck restricting the industrialization of this ethnic medicine system.

This distribution pattern of policy objectives reveals the inherent logic of and practical needs related to the cultural inheritance of and innovations in traditional Chinese medicine (Mongolian medicine). The significant disparities in the proportions of various policy objectives also indicate a tilt in policy priorities. Although this tilt aligns with the needs of the current development stage, from a long-term perspective, the allocation proportions of policy objectives must still be further optimized to achieve development outcomes that are more balanced and sustainable. Particularly in foundational areas such as standardization and theoretical innovation research, policy support should be strengthened to consolidate the developmental foundation and enhance long-term momentum.

### 5.3 Imbalance in the use of policy instruments within policy objectives

The two-dimensional cross-analysis of policy instruments and policy objectives reveals significant discrepancies in their alignment.

The strongest correlation, which obtains between supply-side policy instruments and talent team development, reflects the policy emphasis on talent cultivation. However, this result also demonstrates relatively insufficient support for other policy objectives. The correlation of supply-side policy instruments with scientific research capacity and achievement translation as well as with the development of the health industry ranks next in strength. Their correlation with service capacity, ancient literature compilation and theoretical innovation research, and cultural institution development is relatively high. In contrast, the correlation with international exchange and cooperation as well as standardization projects is the weakest and significantly weaker than is that with talent team development. Such disparities may impede the overall advancement of scientific research and innovation (44).

The distribution of environmental policy instruments across various policy objectives is relatively balanced, but their overall correlation intensity remains low. Environmental policy instruments exhibit the strongest correlation with talent team development, followed by ancient literature compilation and theoretical innovation research. Their correlations with service capacity, scientific research capacity and achievement translation, cultural institution development, and health industry development are moderately high and balanced. The weakest correlation is observed with international exchange and cooperation as well as standardization projects. This “balanced but weak” characteristic may undermine the supportive role of the policy environment.

Demand-side policy instruments are generally weakly correlated with most policy objectives. They are correlated at a relatively high and balanced level with health industry development, international exchange and cooperation, and cultural institution development, whereas their correlation with other objectives is low but balanced. The role of demand-side policy instruments across various objectives is evidently insufficient. Such an imbalance is not conducive to allowing market mechanisms to play a decisive role in resource allocation.

### 5.4 In-depth discussion from the perspectives of health economics and pharmacy administration

From the dual perspectives of health economics and pharmacy administration, combined with the dynamic evolution data of policy instruments from 2016 to 2024 and the current development practice of Mongolian medicine, it can be found that structural imbalance in policy instrument allocation has become a rigid constraint. Among them, financial support accounts for only 2.56%, and standardization projects have a low priority (6.25%). This is not simply insufficient policy investment, but a concentrated reflection of inefficient resource allocation and inadequate institutional supply, which has become the core bottleneck for the marketization of Mongolian medicine and the clinical transformation of in-hospital preparations. A critical and in-depth analysis should be carried out in light of the unique characteristics of Mongolian medicine.

The extremely low proportion of financial support violates the principles of optimal allocation of health resources and cost–benefit theory. The low share of financial allocation essentially reflects the lack of public health fiscal investment in the field of Mongolian medicine. There is neither a stable public financial investment mechanism nor an effective bridge between Mongolian medicine and payers, resulting in insufficient financial support for the inheritance and innovation, standardization construction, and clinical transformation of in-hospital preparations of Mongolian medicine (45). As a distinctive ethnic medicine, the inheritance, innovation, and R&D of in-hospital preparations of Mongolian medicine are characterized by high investment, long cycle, and slow return. The financial allocation ratio of 2.56% is far below the average level of traditional Chinese medicine as a whole. It can neither cover the fixed costs of collating ancient Mongolian medical books and developing characteristic technologies nor support the transformation costs of in-hospital preparations from laboratory to clinical application (46). Such a shortage of funding has invalidated the leverage effect of public financial investment. Social capital is reluctant to participate due to the imbalance between risk and return, forming a vicious cycle: “insufficient investment → lagging innovation → weak marketization”. This not only causes the idleness and waste of characteristic resources of Mongolian medicine but also violates the core goal of health economics—maximizing health output with limited resources—and is seriously inconsistent with the funding demand for the revitalization and development of Mongolian medicine.

The low priority of standardization projects (6.25%) directly disrupts the whole-chain regulation for the clinical transformation of Mongolian medicine in-hospital preparations. The core of pharmacy administration is to ensure drug safety and clinical value through standardization and normalization. At present, Mongolian medicine lacks unified standards for medicinal material quality, preparation technology and efficacy evaluation. As a result, in-hospital preparations have difficulty passing pharmacy approval and filing, cannot be included in the medical insurance payment system, and have limited scope of clinical application. This shortcoming in standardization not only increases the clinical risk of Mongolian medicine preparations but also raises market access barriers, making it difficult for Mongolian medicine to integrate into the mainstream pharmaceutical market and restricting its marketization process. It is also inconsistent with the development orientation of “standardization, normalization, and scientificalization” in China’s pharmacy administration (47) and does not conform to the international trend of standardization in traditional medicine. In addition, the efficiency of pharmacy administration links such as prescription dispensing, quality traceability, and medication guidance for Mongolian medicine in-hospital preparations is low, with hidden dangers in medication safety. Meanwhile, the lack of personalized training targeting the characteristics of Mongolian medicine further restricts the improvement of the standardization level of pharmacy administration for Mongolian medicine (48).

The superimposed effect of the above problems has further aggravated the development dilemma. Insufficient funding leads to a lack of support for standardization construction, while lagging standardization makes it difficult for existing capital investment to achieve effective output. Ultimately, the implementation effect of policies for the inheritance and innovation of Mongolian medicine is greatly weakened. Mongolian medicine can neither fully release its clinical value nor realize high-quality market-oriented and industrial development. This is also the core contradiction that needs to be urgently resolved in the current implementation of Mongolian medicine policies.

## 6 Conclusion

The authors conducted a rigorous content analysis of 30 formal policy documents (encompassing 1,520 coded analysis units) governing the cultural inheritance and innovation of Traditional Chinese Medicine (Mongolian Medicine) in China, issued at the national and Inner Mongolia Autonomous Region levels between 2016 and 2024. Grounded in Rothwell and Zegveld’s seminal typology of supply-side, environment-side, and demand-side policy tools, our analysis advances three interconnected streams of scholarly and practical contribution: theoretical innovation in policy system analysis, methodological advancement in policy text research, and evidence-based actionable implications for policymakers in traditional medicine and cultural heritage governance.

### 6.1 Theoretical contributions

The research’s theoretical contribution lies in its rigorous application and refinement of existing policy analysis frameworks to generate new insights and conceptualizations within the context of traditional medicine policy, thereby offering a robust model for future comparative policy research. The study primarily contributes to policy theory by:

Refining the application of policy tool frameworks: By integrating policy objective dimensions with Rothwell and Zegveld’s policy tool classification (supply-side, environment-side, and demand-side), the research constructs a novel two-dimensional analytical framework. This framework moves beyond a mere categorization of tools to enable a more nuanced understanding of how different policy instruments are aligned with specific policy objectives. This provides a more comprehensive lens for analyzing the effectiveness and structural balance of policy systems in complex fields like traditional medicine.

Highlighting the “Supply-Prioritizes, Demand-Neglects” pattern: The identification of a policy configuration that “prioritizes supply and neglects demand” offers a theoretical insight into potential inherent defects within policy systems aimed at cultural inheritance and innovation, particularly in state-led development contexts. This pattern suggests a theoretical challenge in achieving balanced and sustainable development when market-driving mechanisms are underutilized.

Advancing the understanding of policy imbalance: The study theoretically articulates how structural imbalances within policy instrument categories (e.g., insufficient information support or financial support within supply-side tools) and across policy objectives (e.g., overemphasis on talent vs. underemphasis on standardization) can hinder overall policy effectiveness and long-term sustainability.

### 6.2 Methodological contributions

The study made significant methodological contributions through its approach to policy text analysis:

Development of a robust two-dimensional analytical framework: The construction of a clear and integrated two-dimensional framework (policy tools X policy objectives) provides a replicable and adaptable methodology for analyzing policy texts in other domains. This framework allows for a systematic and quantitative assessment of policy configurations.

Application of NVivo for coding and quantitative analysis: The use of NVivo 15 software for coding 1,520 analysis units from 30 policy documents demonstrates a rigorous and systematic approach to content analysis. This quantitative method provides empirical data to support the identification of policy patterns and imbalances, enhancing the objectivity and reliability of policy text analysis.

Cross-analysis technique for instrument-objective alignment: The two-dimensional cross-analysis technique employed to reveal the correlation strength between policy instruments and policy objectives offers a powerful method for diagnosing discrepancies and identifying areas of misalignment within a policy system. This goes beyond simply listing tools and objectives to show their interactive dynamics.

### 6.3 Practical implications

#### 6.3.1 Systematic optimization of policy instrument structure

On the basis of the analysis of the aforementioned issues, the policy system for the cultural inheritance of and innovations in traditional Chinese medicine (Mongolian medicine) should be optimized systematically, involving in-depth adjustments at both the overall layout and the internal structure levels of policy instruments.

In terms of the overall layout, the proportional relationships must be restructured among supply-side, environmental, and demand-side policy instruments. The proportion of supply-side policy instruments should be moderately decreased, and the proportion of demand-side policy instruments should be increased. Although the Mongolian medicine sector currently relies on state subsidies and infrastructure construction, supply-side instruments exhibit a structural imbalance: non-core instruments (e.g., general infrastructure) account for an excessively high proportion, while core inheritance instruments (e.g., ancient literature collation, research translation) are underutilized. A moderate reduction aims to optimize the policy structure, rather than cut core support. This structural adjustment aims to achieve a new configuration of policy instruments characterized by “moderate supply, an optimized environment, and increased demand”.

With respect to the structural optimization of supply-side policy instruments, the degree of institutional infrastructure development should be adjusted to avoid redundant construction and resource waste. Simultaneously, the proportion of document collection and preservation should be increased significantly to strengthen the protection of traditional cultural resources. The proportion of financial support also needs to be increased to enhance the substantive effectiveness of policies. With respect to scientific and technological support and talent cultivation, their proportions should be maintained within reasonable ranges, but greater emphasis should be placed on strengthening the synergy between the two to foster a more closely integrated, interactive relationship.

Structural adjustments to environmental policy instruments should focus on optimizing the proportion of policies and regulations. Their proportion should be decreased moderately while the precision and effectiveness of policies should be improved. On this basis, the proportion of incentive mechanisms should be increased to strengthen the endogenous motivation of market entities (49). The proportion of multiparty collaboration mechanisms also needs to be enhanced to promote effective interactions among various stakeholders. With respect to goal planning as well as supervision and assessment, reasonable proportions should be maintained, but greater effort should be directed toward improving their scientific rigor and operability to ensure that policy outcomes can be realized effectively.

Strengthening the structure of demand-side policy instruments should involve innovating support methods and enhancing the focus and effectiveness of policies while maintaining the dominant role of industrial promotion and support. Policy support should be further intensified, as an increase in its proportion can enhance its practical impact. The demonstrative and leading role also needs to be strengthened, with a recommended proportion increase to > 4% to leverage the driving effect of exemplary models. With respect to international exchange and cooperation, a reasonable proportion of 5–6% should be maintained, but cooperation mechanisms should be optimized to increase the level of international development.

#### 6.3.2 Optimization and improvement of the policy objective system

The policy objective system should be optimized by focusing on achieving balance, synergy, and feasibility among the objectives. With respect to the hierarchical structure of objectives, the proportion allocated to talent team development should be moderately reduced to avoid an excessive concentration of resources. Simultaneously, the proportion allocated to ancient literature collation and theoretical innovation research should be increased significantly to solidify the developmental foundation. Support for standardization projects also needs to be strengthened to promote the standardized development of the industry. For the three key areas—service capacity, health industry development, and scientific research capability with achievement translation—reasonable proportions should be maintained. However, greater emphasis should be placed on enhancing the interactive relationships among them to foster a mutually reinforcing development dynamic.

In terms of objective synergy mechanisms, a scientific evaluation mechanism for interobjective linkage should be established to ensure the coordinated advancement of all objectives. Additionally, a tiered progression system should be developed for achieving the objectives, ensuring organic alignment between short-, medium-, and long-term goals to avoid fragmentation and short-termism in policy objectives. Furthermore, a comprehensive evaluation system for objective assessment should be improved to prevent policy deviations caused by single-indicator evaluations and holistically realize policy objectives.

#### 6.3.3 Pathway optimization for matching policy instruments and objectives

To enhance the alignment between policy instruments and objectives, a scientific categorical mapping mechanism should be established. Doing so involves creating a dynamic matching matrix for policy instruments and objectives to align them precisely. With respect to key objectives at different development stages, appropriate combinations of policy instruments should be configured. Concurrently, a monitoring and evaluation mechanism should be established for the effectiveness of policy instrument usage so the instrument mix can be established in a timely manner (50). With respect to collaborative implementation mechanisms, an interdepartmental policy coordination mechanism should be developed to avoid policy fragmentation, strengthen complementarity among policy instruments, and improve the overall effectiveness of policies (51).

In terms of resource-allocation mechanisms, a scientific priority sequence for policy resources should be established to ensure that core objectives are achieved. Differentiated instrument combinations should be formulated according to regional resource endowments and economic levels, with the integration of market-oriented concepts. Demand-side instruments should be adopted to stimulate the participation of market entities, so as to achieve coordinated efforts between the government and the market, which is consistent with the development direction of China’s cultural policies (52). Simultaneously, the market-oriented mechanism for resource allocation should be refined to enhance resource utilization efficiency (53). Methods of policy support should be innovated to strengthen the sustainability of policies. Furthermore, supporting measures such as organizational, institutional, technical, and financial safeguards should be established and improved to ensure the effective implementation of the proposed policy optimizations.

Through these systematic optimization measures, the scientific rigor and effectiveness of the policy system for the cultural inheritance of and innovations in traditional Chinese medicine (Mongolian medicine) can be effectively enhanced, which would provide stronger policy support for the industry’s sustained and healthy development. These optimization suggestions should be implemented gradually, balancing policy stability and continuity while making timely adjustments based on actual conditions to maximize the effectiveness of policy improvements. Additionally, the decisive role of the market in resource allocation should be fully leveraged to mobilize the initiative of various market entities, thereby fostering a new pattern of policy implementation that combines government guidance with market-driven dynamics.

Drawing on the structural imbalances in Mongolian medicine policy instruments identified in this study, this paper proposes an integrated three-dimensional collaborative implementation framework to address the persistent practical dilemma of unenforceable policies and inefficient input–output translation. This framework comprises three interlocking core components: a linkage mechanism between medical insurance access for clinically proven Mongolian medicine in-hospital preparations and targeted grassroots talent training, a demand-driven collaborative chain of specialized procurement access and industrial support for Mongolian medicine products, and a cross-departmental joint meeting system for dynamic policy evaluation and adaptive adjustment. The core design of this framework is to construct a closed-loop policy system: it leverages demand-side instruments represented by medical insurance payment to unlock market potential, empowers clinical service capacity through supply-side instruments centered on targeted talent training, and strengthens cross-sectoral implementation via environmental-side institutional design, with the ultimate goal of facilitating the high-quality inheritance, innovation and sustainable development of Mongolian medicine.

### 6.4 Limitations of the study and future research directions

While the research provides a comprehensive and systematic analysis of traditional medicine policy design through a policy tool lens, it is subject to several notable limitations that define the scope of its findings, while also illuminating meaningful avenues for future academic inquiry.

First, this study is limited to *de jure* content analysis of formal policy documents, focusing only on the stated intent and structural design of policies, without investigating their *de facto* implementation, real-world effectiveness, on-the-ground impacts, or stakeholder experiences. Therefore, the identified policy imbalances and structural shortcomings are derived solely from policy design, rather than empirical evaluation of actual implementation outcomes.

Second, the narrow geographical and temporal scope of the sample restricts the generalizability of the findings. This study only includes national and Inner Mongolia Autonomous Region policy texts issued between 2016 and 2024, excluding regional policies from other Mongolian-inhabited provinces in China, informal policy guidance, project-specific initiatives, pre-2016 foundational policies, and the latest policy trends. While Inner Mongolia’s policy system is highly representative of China’s Mongolian medicine governance as the core development region of the sector, the exclusion of other regional practices limits the nationwide applicability of the conclusions.

Third, the analytical approach has inherent constraints. The exclusive reliance on Rothwell and Zegveld’s single policy tool framework may restrict the nuance of the analysis, as alternative policy typologies could reveal complementary dimensions of policy intervention. Meanwhile, the core focus on the frequency and proportional distribution of policy instruments fails to capture the qualitative intensity, strategic weight, or actual resource allocation of each policy element, potentially oversimplifying the complex interplay within the policy system. In addition, despite strict quality control measures including double independent coding (Cohen’s Kappa = 0.9504) and expert review, the qualitative coding process inevitably carries subjective judgment, an inherent limitation of content analysis. Finally, this study focuses on static structural analysis of policy texts, and does not use rigorous causal inference methods to examine the impact of key policy events, with the dynamic evolution mechanism of Mongolian medicine policies remaining to be explored in in-depth longitudinal studies.

Building on these limitations, multiple promising directions for future research have emerged: scholars can conduct empirical evaluations of policy implementation and impact, combining qualitative stakeholder interviews and quantitative econometric assessment to unpack the gaps between policy intent and real-world outcomes and identify barriers to effective policy delivery; expand the geographical and comparative scope of analysis, both to explore regional variations in policy design across Chinese provinces and to conduct cross-national comparative research using the adapted two-dimensional analytical framework in other countries with mature traditional medicine systems; integrate multiple policy tool typologies to develop a more holistic, hybrid analytical framework tailored to the unique complexities of traditional medicine and cultural heritage governance; move beyond frequency-based quantitative analysis to conduct in-depth qualitative content analysis that explores the rhetorical framing, underlying political economy, and philosophical orientation of traditional medicine policy; and undertake longitudinal studies to track the long-term evolution of policy priorities and the dynamic alignment between policy tools and strategic objectives over extended timeframes, all of which will build on the foundational contributions of this study to advance a more comprehensive, nuanced understanding of traditional medicine policy governance.
